# Supplementary figures and images for: A Murine Model of Glioblastoma Initiating Cells and Human Brain Organoid Xenograft for Photodynamic Therapy Testing
Source: Int J Mol Sci. 2025 Sep 12;26(18):8889. doi: 10.3390/ijms26188889 (PMC12470220; doi:10.3390/ijms26188889)

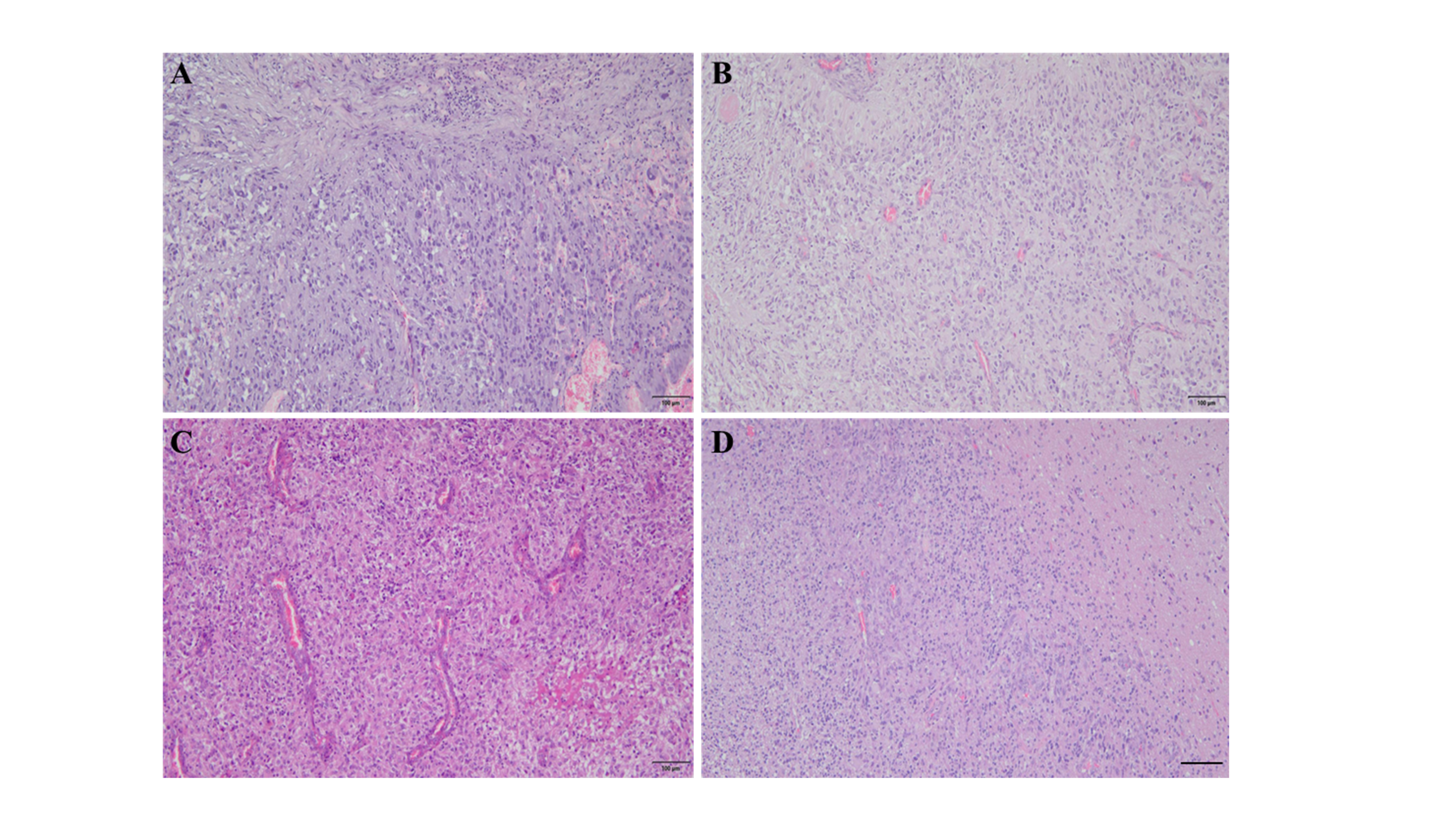

Supplement: Supplementary file 1 [file ijms-26-08889-s001.zip › Fig S1.tif]

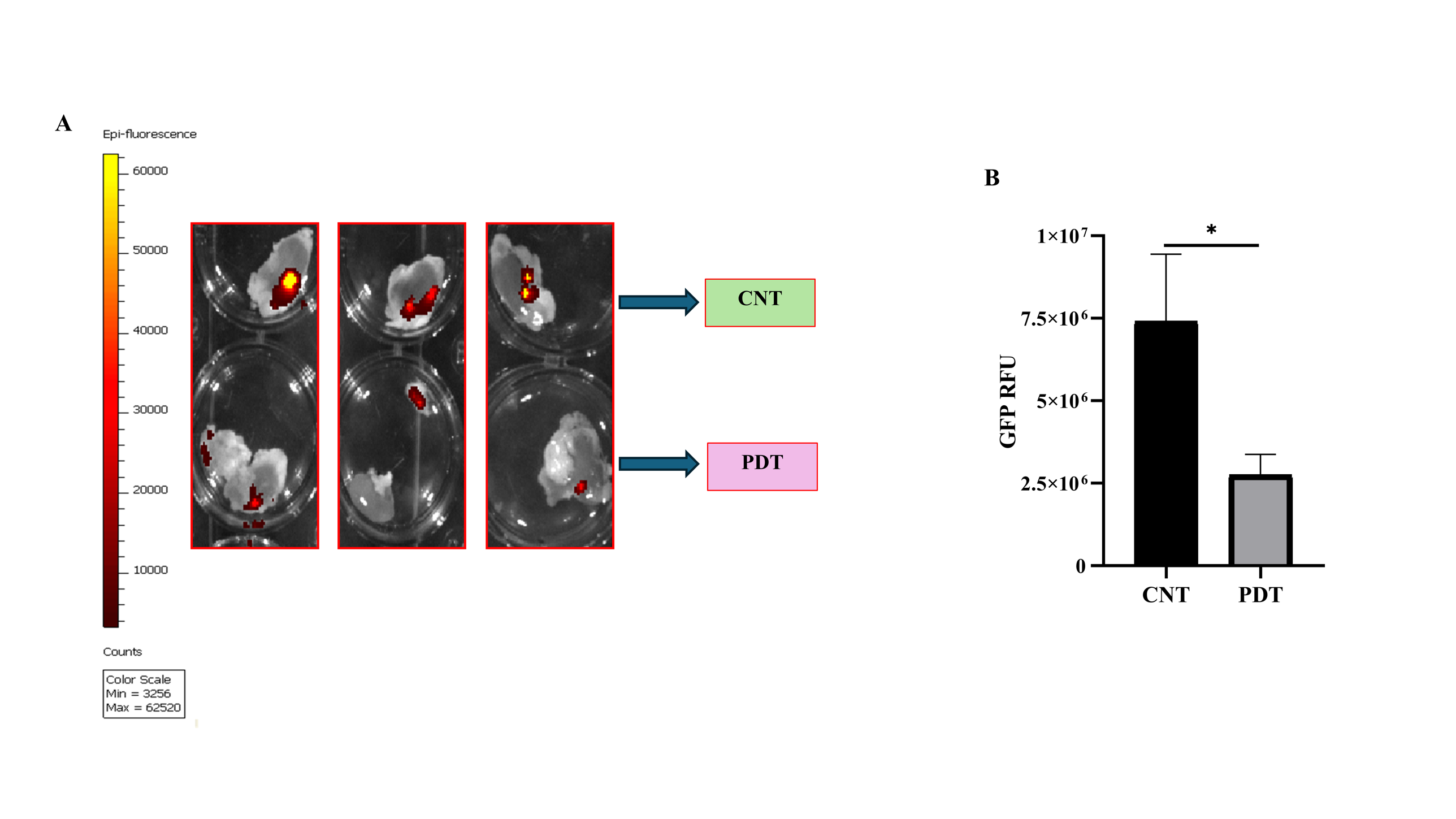

Supplement: Supplementary file 1 [file ijms-26-08889-s001.zip › Fig S10.tif]

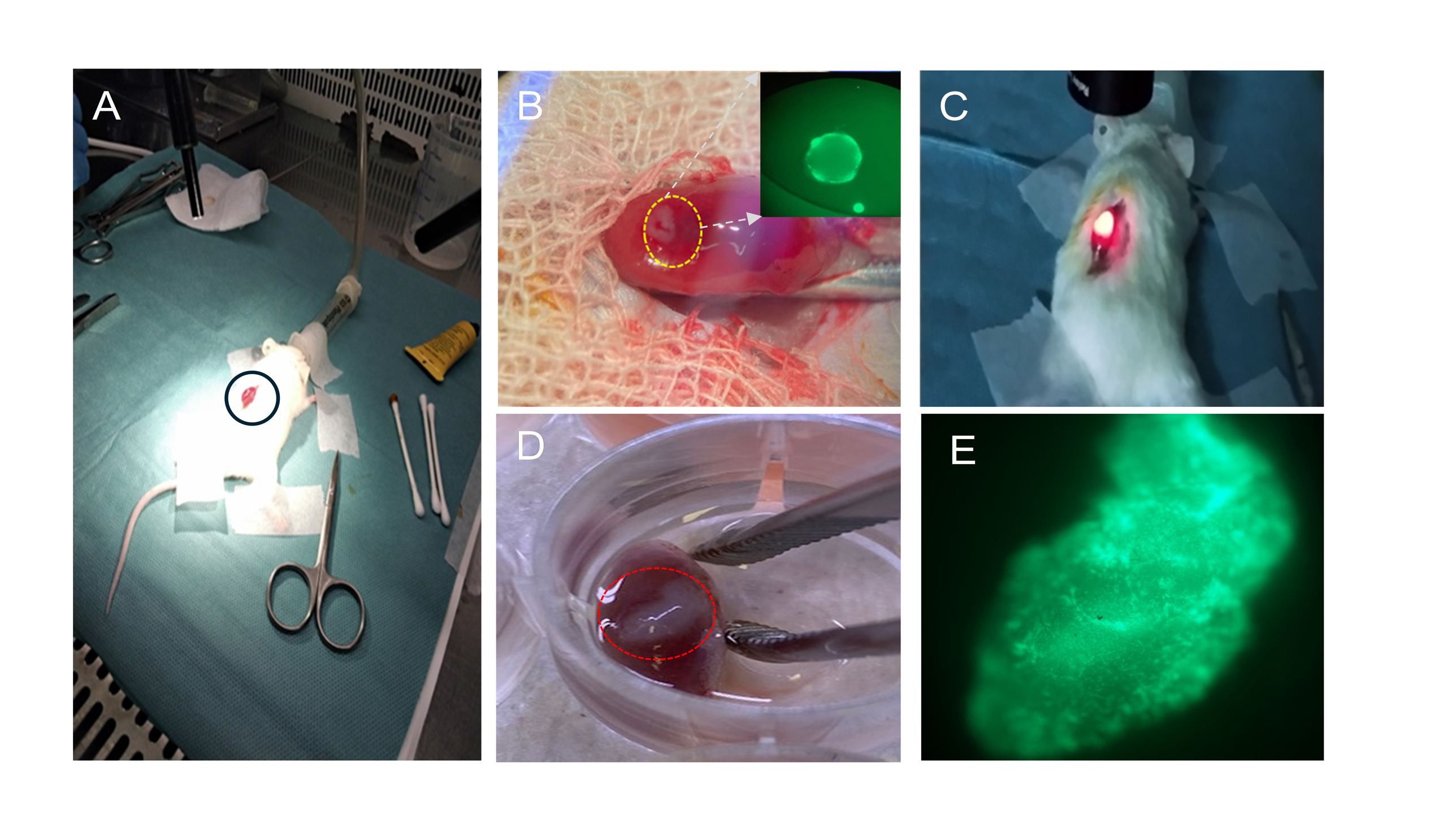

Supplement: Supplementary file 1 [file ijms-26-08889-s001.zip › Fig S11.tif]

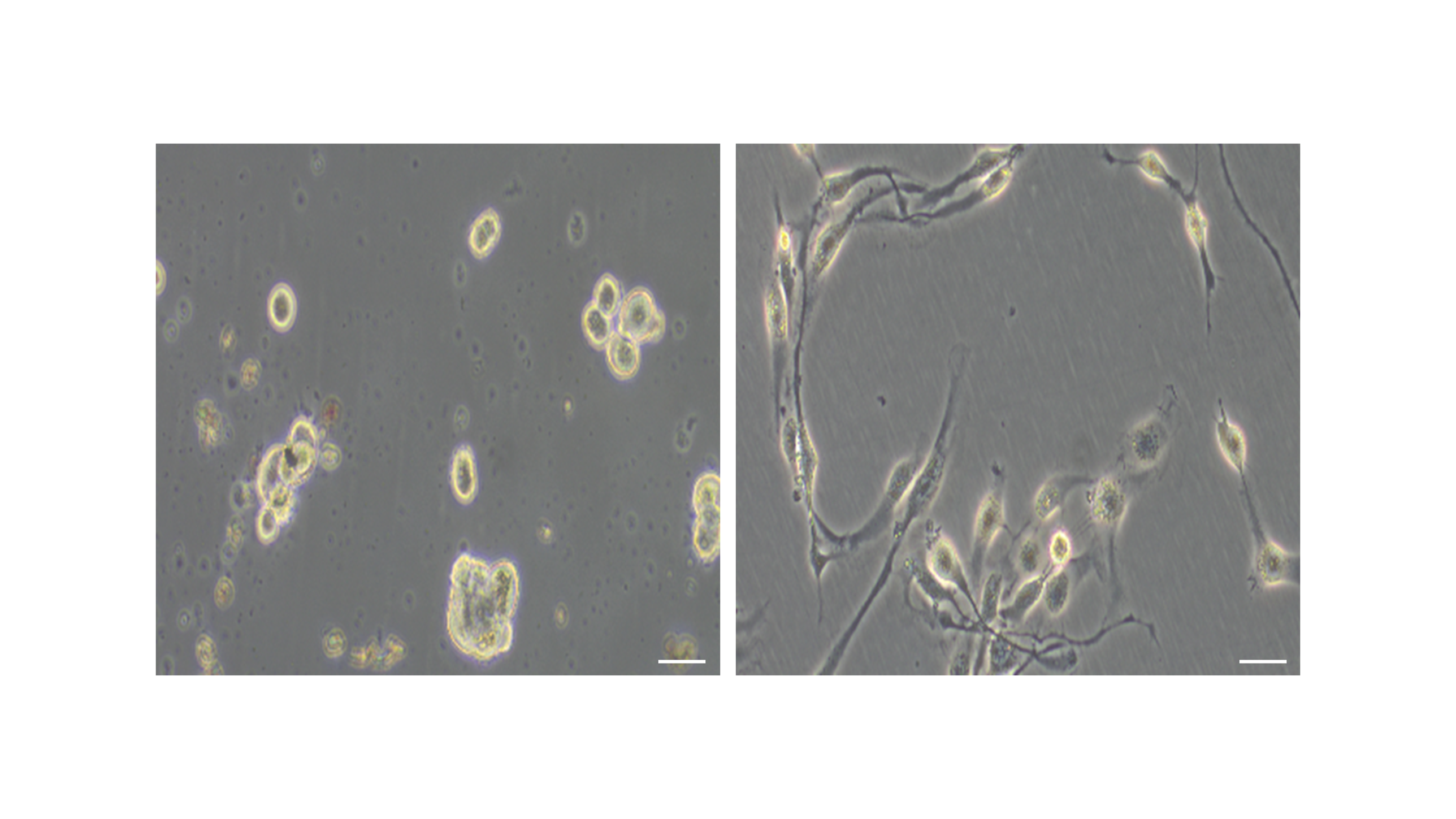

Supplement: Supplementary file 1 [file ijms-26-08889-s001.zip › Fig S2.tif]

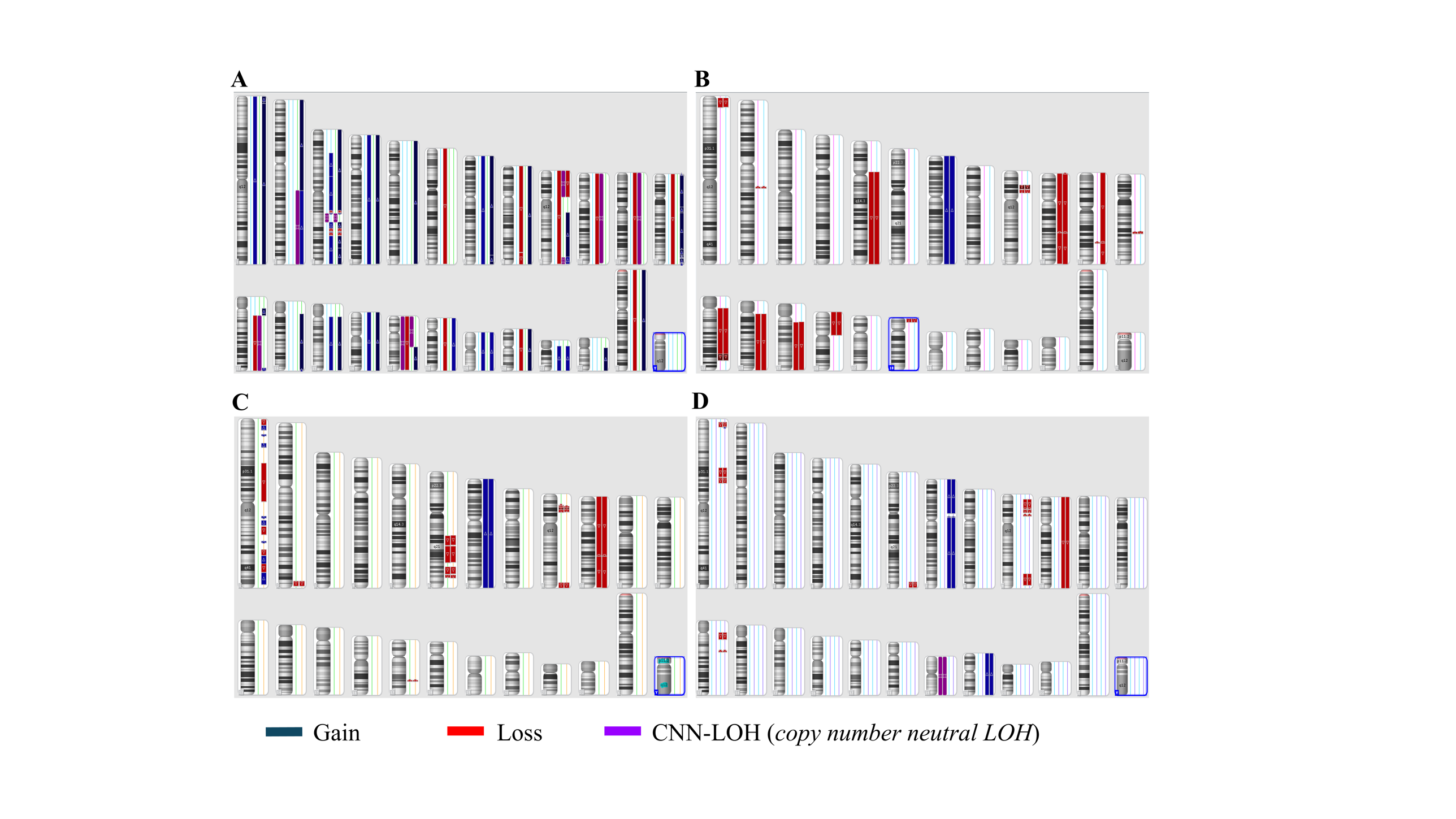

Supplement: Supplementary file 1 [file ijms-26-08889-s001.zip › Fig S3.tif]

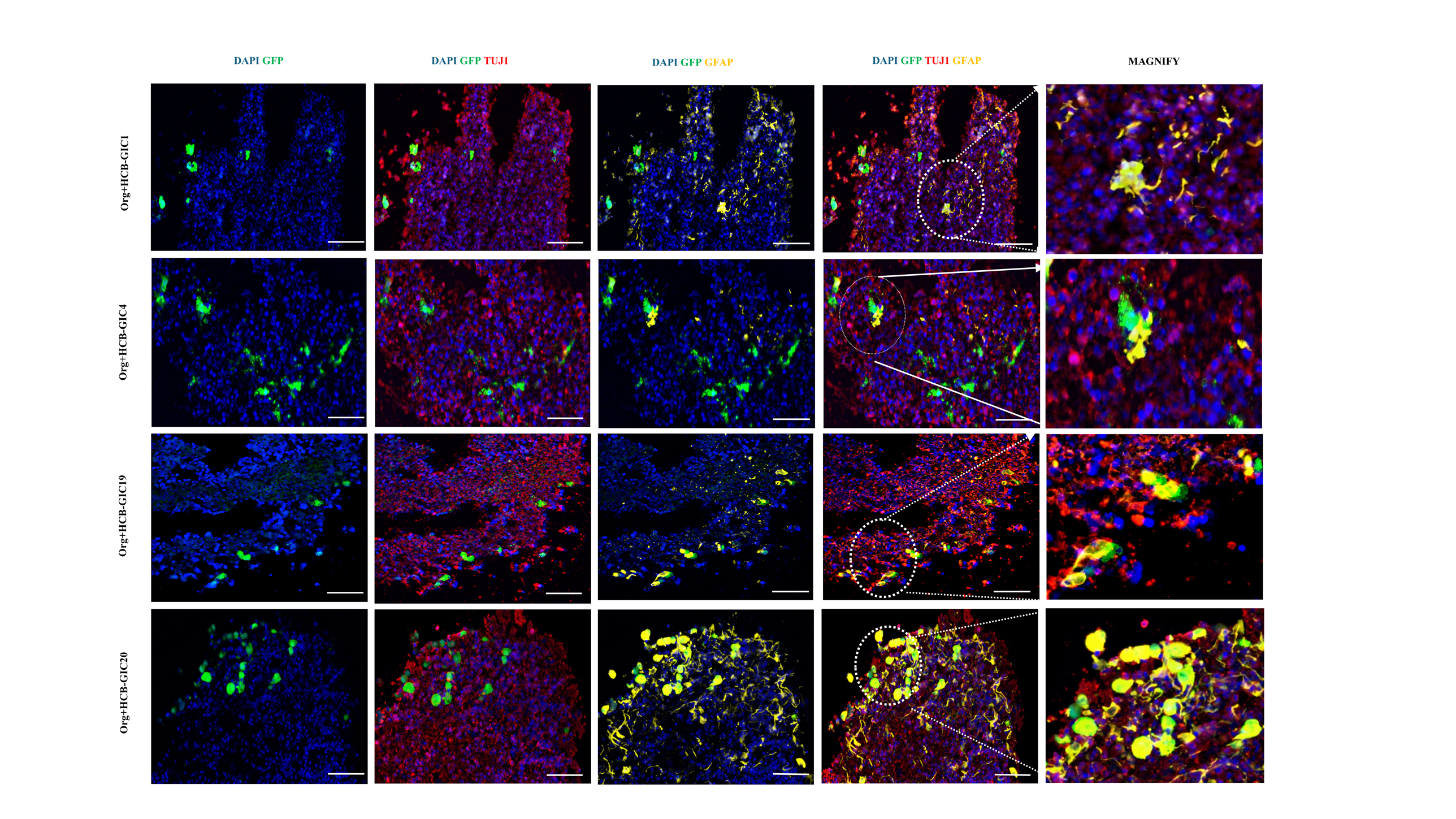

Supplement: Supplementary file 1 [file ijms-26-08889-s001.zip › Fig S4.tif]

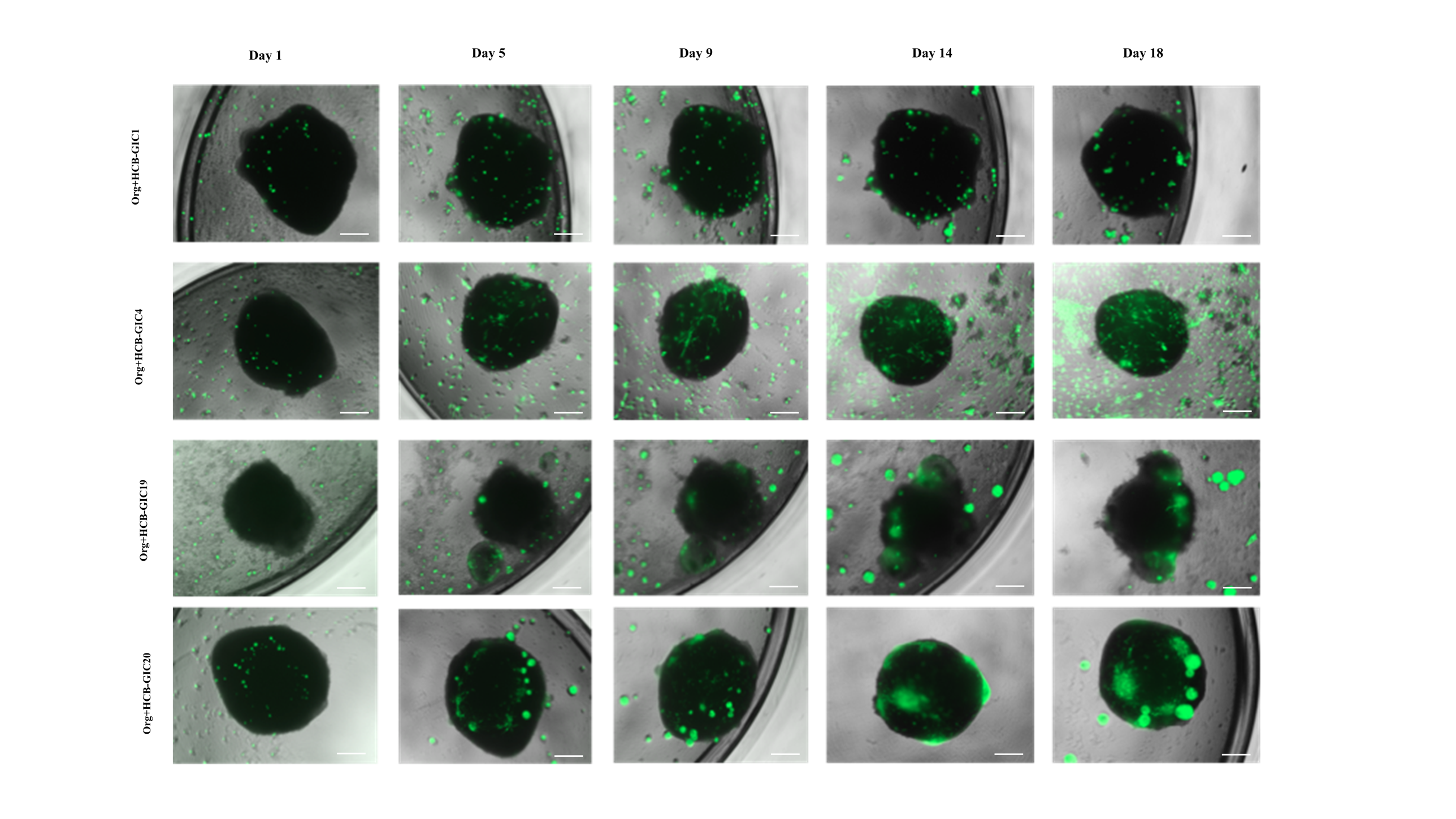

Supplement: Supplementary file 1 [file ijms-26-08889-s001.zip › Fig S5.tif]

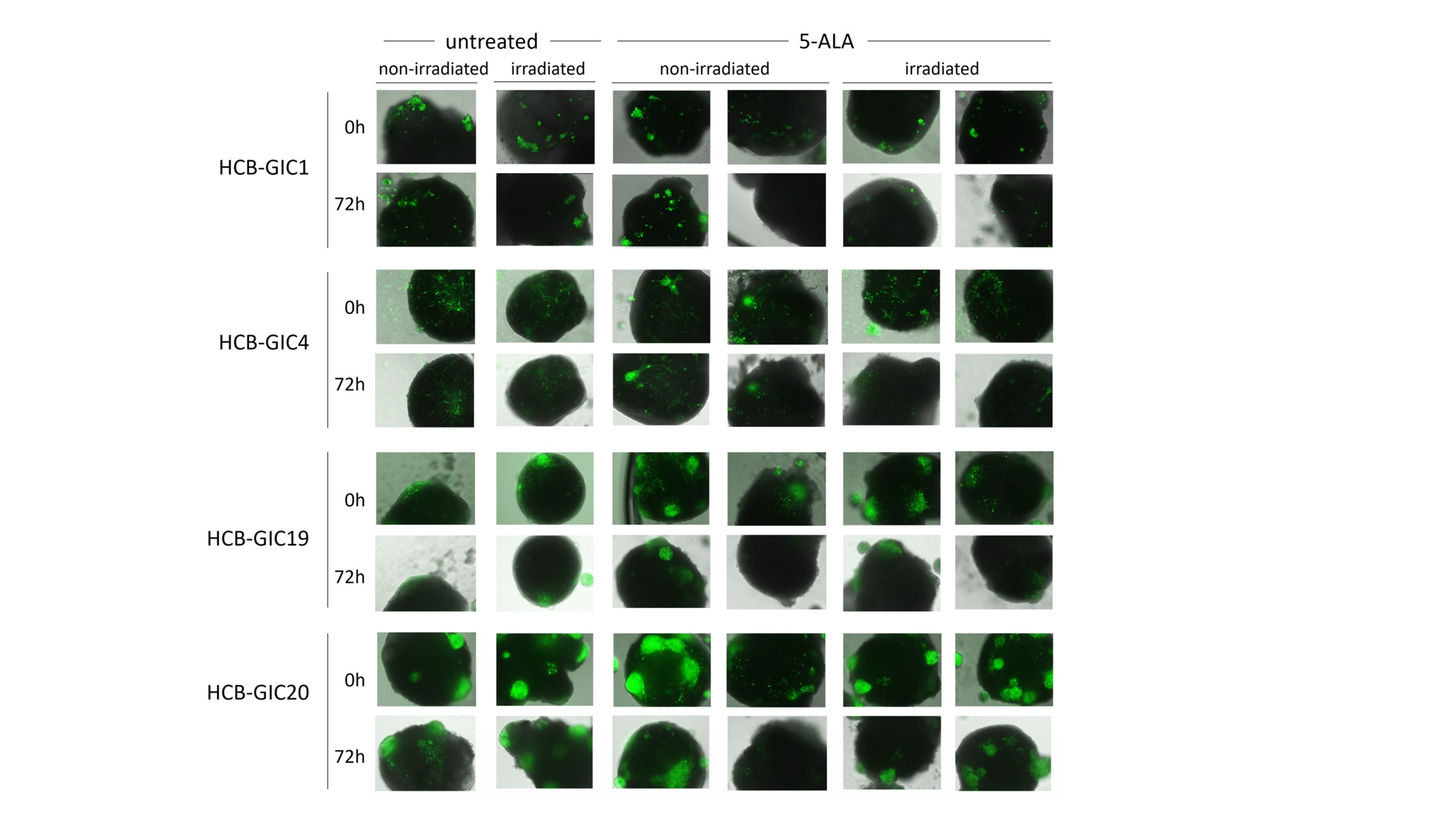

Supplement: Supplementary file 1 [file ijms-26-08889-s001.zip › Fig S6.tif]

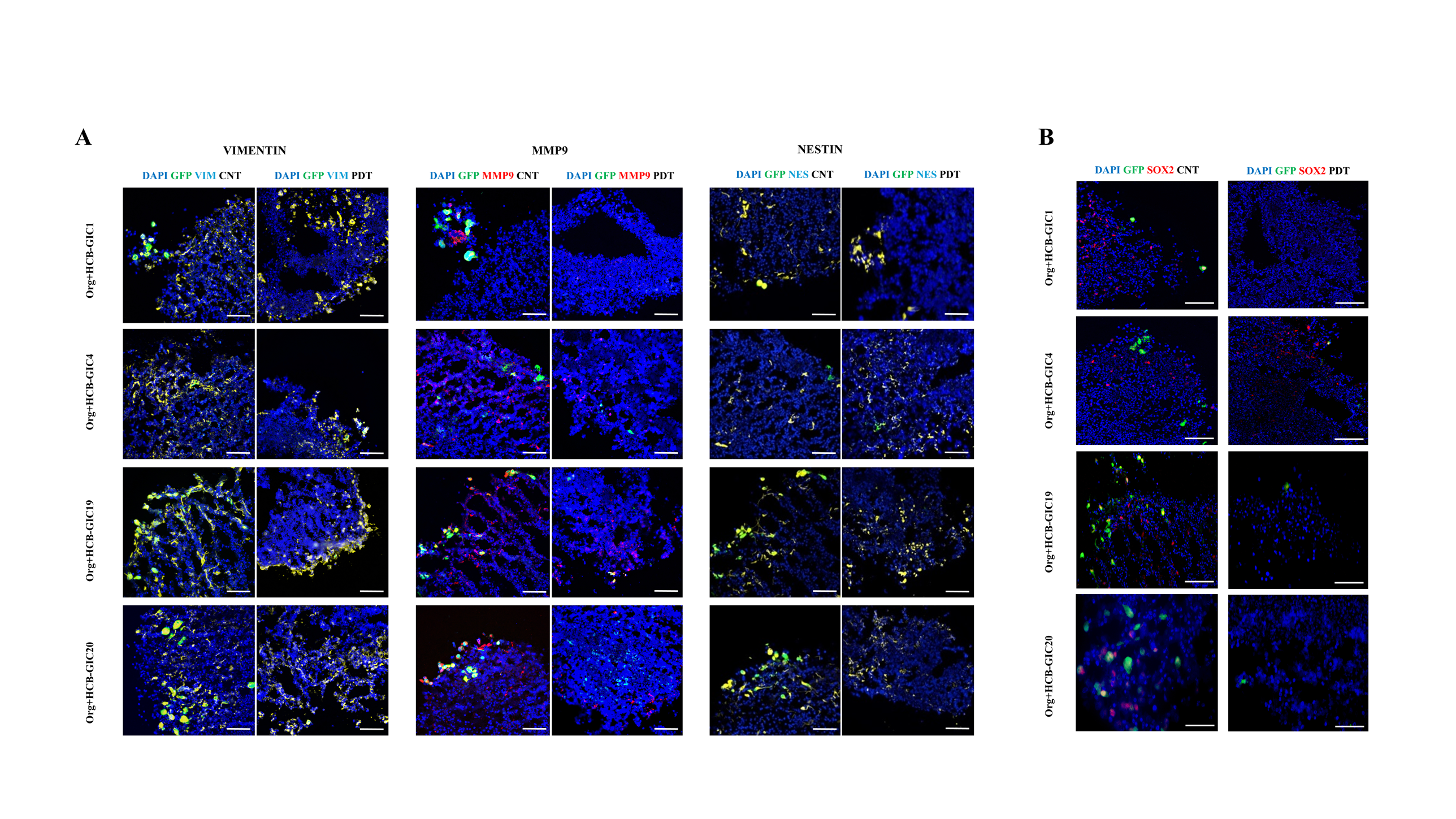

Supplement: Supplementary file 1 [file ijms-26-08889-s001.zip › Fig S7.tif]

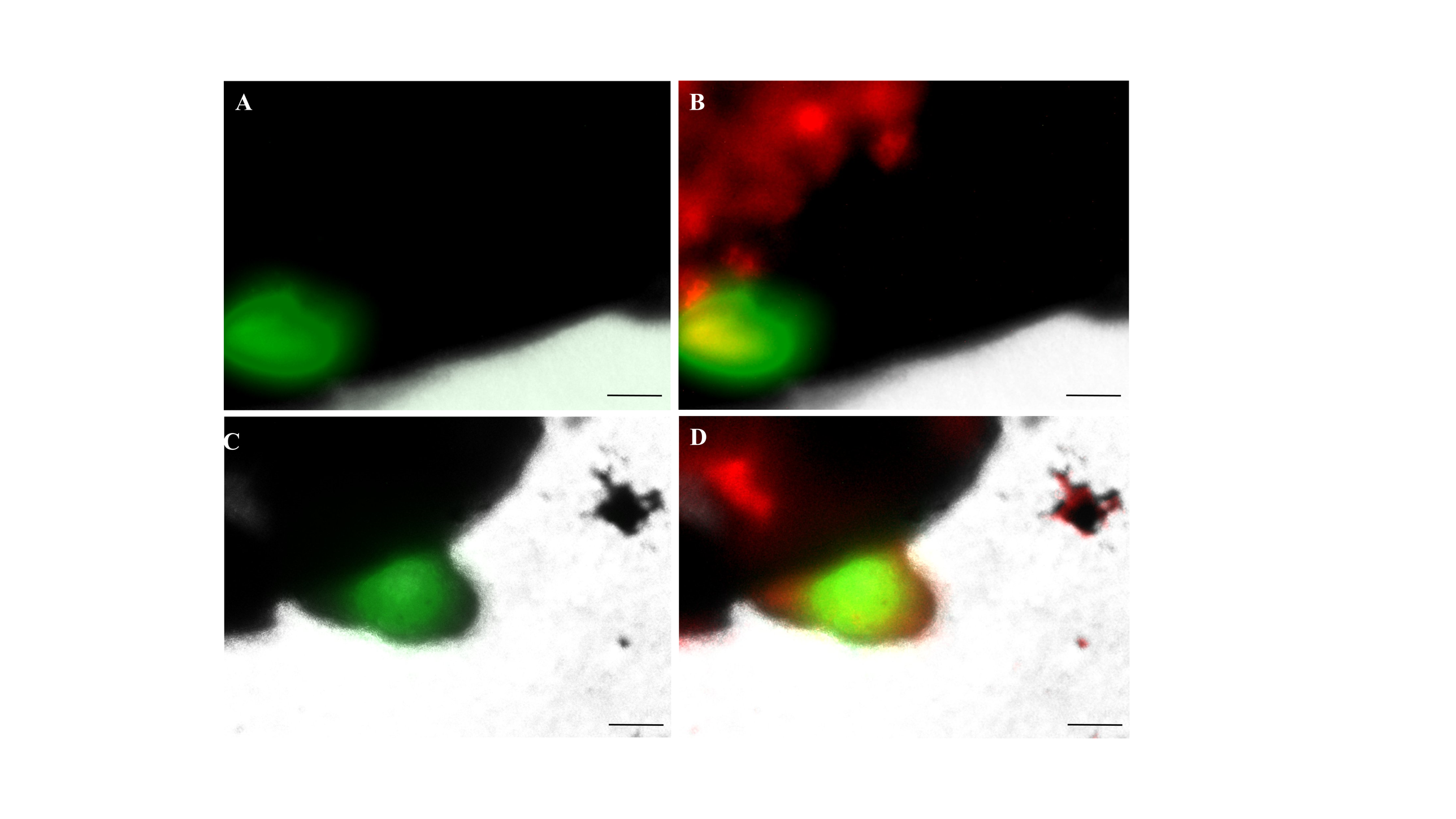

Supplement: Supplementary file 1 [file ijms-26-08889-s001.zip › Fig S8.tif]

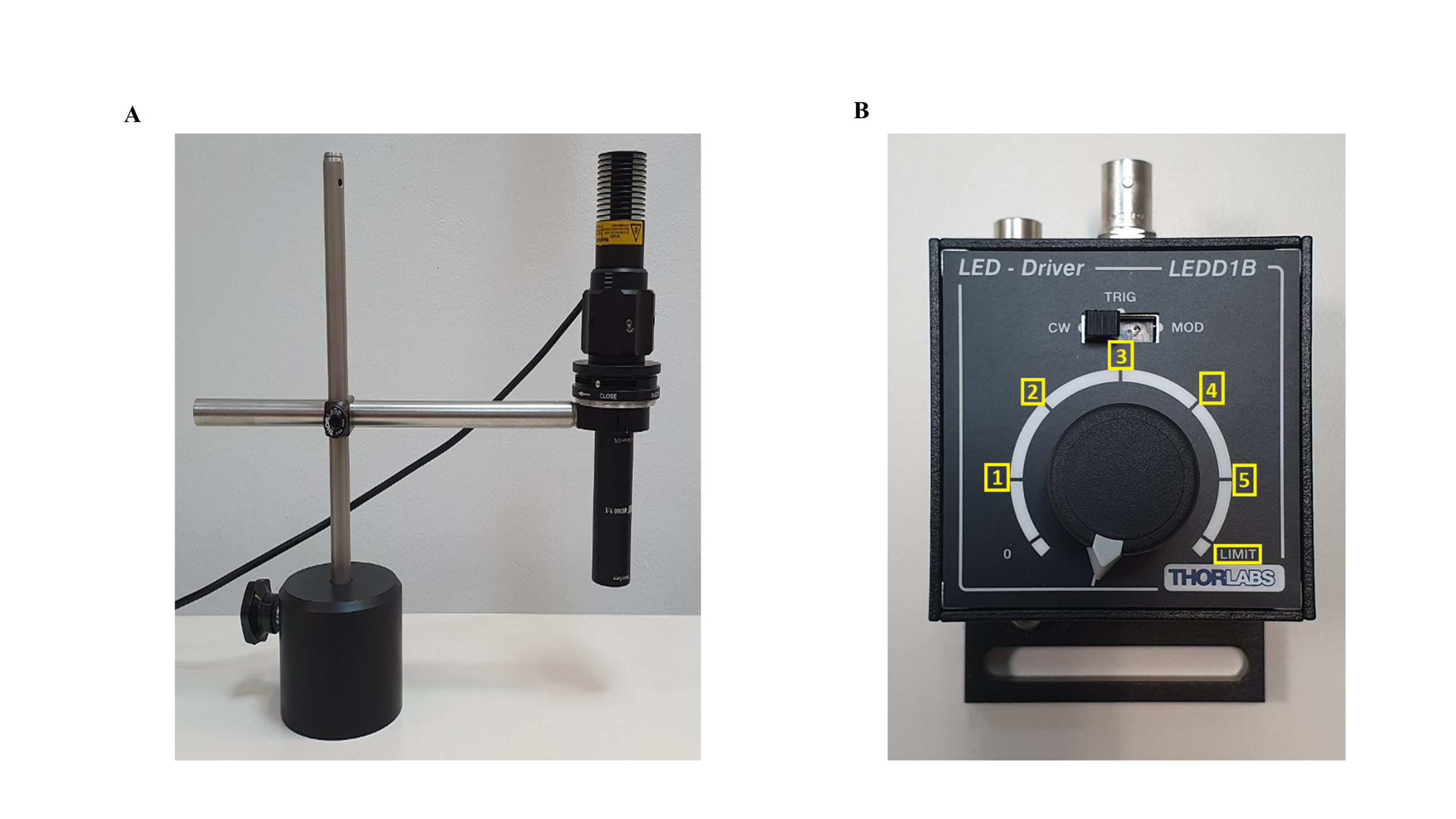

Supplement: Supplementary file 1 [file ijms-26-08889-s001.zip › Fig S9.tif]
